# Improving adjustments for older age in pre-hospital assessment and care

**DOI:** 10.1186/1757-7241-21-4

**Published:** 2013-01-23

**Authors:** Marius Rehn

**Affiliations:** 1 Department of Research, Norwegian Air Ambulance Foundation, P. O. Box 94, Drøbak, 1448, Norway; 2 Department of Anaesthesia and Intensive Care, Akershus University Hospital, Lørenskog, Norway

## Abstract

Population estimates projects a significant increase in the geriatric population making elderly trauma patients more common. The geriatric trauma patients experience higher incidence of pre-existing medical conditions, impaired age-dependent physiologic reserve, use potent drugs and suffer from trauma system related shortcomings that influence outcomes. To improve adjustments for older age in pre-hospital assessment and care, several initiatives should be implemented. Decision-makers should make system revisions and introduce advanced point-of-care initiatives to improve outcome after trauma for the elderly.

## An ageing population

**Figure 1:**
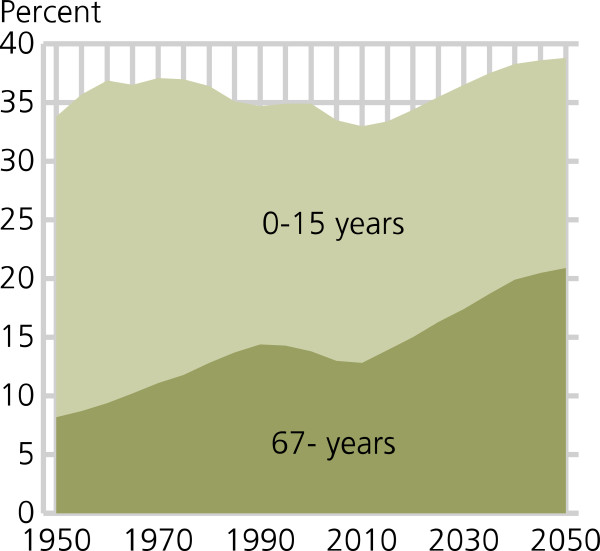
**Percentage of children/young people and elderly in the Norwegian population.** Source: Statistics Norway www.ssb.no (permission for reprint granted).

Optimal trauma care relies on decision-makers that dynamically accommodate emergency medical services (EMS) to national trends, including demographical variations. Several population estimates indicate increased life expectancy and growth in population size. Statistics Norway projects a significant increase in population proportion above 67 years of age, pictorially calling it *“the grey tsunami”* (c.f. Figure 1 for demographic projections) [1]. A similar trend is seen in UK where the population is ageing and is projected to continue ageing over the next decades [2]. As a consequence, our patients are getting older making geriatric trauma more common.

## Extremes of age; geriatric trauma patient

EMS providers must adjust for the changes that occur in patients at extremes of age. While children have different baseline vital signs and airway anatomy, elderly people have sensory changes that result in deviating physiological responses and manifestations of shock [3,4]. The pattern of injuries among geriatric patients is predominated by blunt trauma such as falls, motor vehicle collisions and pedestrian events [5-7]. Unsurprisingly, there is a positive correlation between increasing age and increasing incidence in pre-existing medical conditions [8,9]. Pre-existing co-morbidity has been associated with an age-independent increase in mortality after trauma. Skaga and colleagues found that pre-injury co-morbidity scored according to the American Society of Anaesthesiologists (ASA) classification system was an independent predictor of trauma mortality [10,11]. Hollis and colleagues found that pre-existing co-morbidity was associated with increased mortality when combined with low to moderate severity trauma, but not when combined with more severe trauma [12].

Further, the geriatric trauma population is a more frequent user of medication that influence outcome after injury. Anticoagulants increase the risk for intracranial haemorrhage and longer hospital stays following head injury [13,14]. Further, beta-blockers may mask tachycardia introducing bias in the interpretation of vital signs following injury [4].

Lastly, elderly trauma victims are exposed to age-related trauma system bias. Chang and colleagues found that increasing age was associated with increased undertriage [15]. They also conducted a follow-up survey of the EMS personnel involved. The survey revealed inadequate training, unfamiliarity with triage protocol, and age bias to be causal factors for the age-dependent undertriage.

## Death, disease, destitution

The geriatric trauma patients are exposed to several risk factors: higher incidence of pre-existing medical conditions, impaired age-dependent physiologic reserve, potent drugs and trauma system related shortcomings. How does this influence outcome?

Kuhne and colleagues found mortality to increase at 56 years and that the increase was independent of injury severity [16]. Søreide and colleagues found that elderly patients are more at risk for multi organ failure (MOF) and that MOF occur despite low injury severity [17]. Other studies indicate that elderly patients have higher complication rates such as infections compared to younger cohorts and that they die later in their admissions [18,19]. Accordingly, elderly patients have poorer outcomes and consume disproportionate amount of resources due to frequent admissions and lengthier stays at intensive care units.

## Improvement initiatives

**Figure 2:**
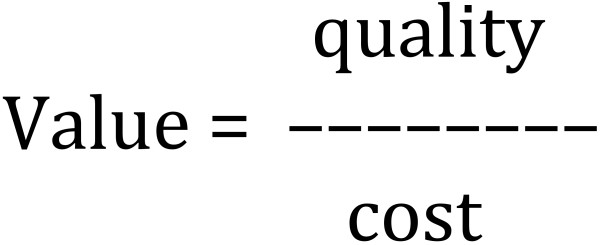
Value equation. Increase value: Increase quality and/or decrease cost.

The *“grey tsunami”* poses a challenge to clinical decision-makers as it creates a larger demand for already scarce pre-hospital resources. However, increased EMS funding alone cannot meet this challenge. Clinical leaders must launch quality improvement initiatives to increase the value of their services (c.f. List of suggested improvement initiatives) [20]. By increasing the numerator (quality) while maintaining the denominator (cost), the value will increase (c.f. Figure 2 for value equation) [21].

List of suggested improvement adjustments

•Quality improvement initiatives

•Update field triage guidelines

•Field access to national patient core record

•Field access to digital prescription system

•Termination of resuscitation rules

•Tiered trauma team activation

•Geriatric consultation service as add on to the trauma team

•Field access to arterial blood gas analyses

•Field access to radiological evaluations

## System revisions

Newgard and colleagues found that triage criteria loose sensitivity at the ends of age spectrum, introducing the need for separate elderly-specific criteria [22]. Heffernan and colleagues found that mortality significantly increases with systolic blood pressure (SBP) <110 mmHg in the geriatric patients but not until a SBP of 95 mmHg in the younger cohorts. Chisholm and colleagues found that approximately 35% of all deaths among patients aged ≥65 years were attributed to ground level falls [23]. A combined literature review and US national expert panel consensus resulted in the 2009 “Guidelines for Field Triage of Injured Patients” [24,25]. In the 2011 revision of these field triage guidelines, two criteria addressing geriatric trauma have been added: “SBP <110 might represent shock after age 65 years” and “Low-impact mechanisms (e.g., ground-level falls) might result in severe injury” [26]. Accordingly, decision makers should implement updated evidence-based field triage guidelines to reduce age-related mistriage.

EMS providers should establish pre-injury ASA to adjust for pre-existing medical conditions in their evaluations of geriatric trauma victims [11]. Implementing field access to a national patient core record would facilitate co-morbidity adjustments improving triage accuracy among the elderly. Further, field access to a digital prescription system would inform EMS providers of medication use that influence further management.

Wuerz and colleagues found that age has less effect on resuscitation success than other factors such as early cardio pulmonary resuscitation and advanced life support. They concluded that high age alone should not deter resuscitation attempts. However, to reduce uncertainness in end-of-life considerations in geriatric patients, EMS providers should have access to evidence-based termination of resuscitation rules [27].

A tiered approach with a reduced trauma team has proven to reduce the threshold for trauma team activation while avoiding unnecessary resource consumption [28]. Tiered trauma teams are recommended by the US National guidelines for field triage and should be considered as an improvement adjustment to accommodate the increasing number of elderly trauma victims [26]. Further, a geriatric consultation service as add on to the trauma team has been recommended [29]. These experts may optimize pre-existing medical conditions and manage delirium already in the initial phase of in-hospital resuscitation.

## Point-of care diagnostics

Martin and colleagues found that occult hypoperfusion as determined by base deficit and lactic acid was present in 42% of geriatric patients with “normal” vital signs [30]. Field access to arterial blood gas analyses may improve the point-of-care diagnostic accuracy, conversely contributing to improved triage and treatment initiatives.

Elderly patients with impaired consciousness may prove a diagnostic challenge to EMS providers. The Norwegian Air Ambulance Foundation is currently implementing pre-hospital computer tomography units with telemedical support [31]. Such integrated solutions of advanced point-of-care diagnostics may identify traumatic brain injury or cerebral ischemia caused by either haemorrhage or infarction. Advanced point-of-care diagnostics may facilitate neuron-saving therapy already in the field. Although the feasibility of such advanced interventions remains uncertain, decision-makers should include EMS in the technological development seen in other areas of medicine. By combining low-tech system revisions with high-tech diagnostics, we may improve our adjustments for older age in pre-hospital assessment and care.

## Abbreviations

EMS: Emergency Medical Services; ASA: American Society of Anaesthesiologists; MOF: Multi Organ Failure; SBP: Systolic Blood Pressure.
